# The Effects of Mental Fatigue on Psychophysiological Responses, Mood States, and Archery Shooting Performance Under a Simulated Archery Competition: A Randomized Cross-Over Study

**DOI:** 10.3390/brainsci16050459

**Published:** 2026-04-24

**Authors:** Sevval Soylu, Ersan Arslan, Bulent Kilit, Yusuf Soylu

**Affiliations:** 1 Department of Movement and Training, Institute of Graduate Education, Tokat Gaziosmanpasa University, Tokat 60250, Türkiye; sevvalssoylu@gmail.com; 2 Faculty of Sports Sciences, Tokat Gaziosmanpasa University, Tokat 60250, Türkiye; bulent.kilit@gop.edu.tr (B.K.); yusuf.soylu@gop.edu.tr (Y.S.)

**Keywords:** cognitive load, target shooting, Stroop task, physiological responses, compound-bow archers

## Abstract

**Highlights:**

**What are the main findings?**

- Mental fatigue significantly impaired archery shooting performance and increased perceived exertion and mental effort despite no changes in heart rate.
- Mental fatigue alters mood states (increased anger and fatigue) and reduces enjoyment, indicating a strong psychophysiological impact without parallel physiological responses.

**What are the implications of the main findings?**

- Mental fatigue primarily affects perceptual-cognitive and affective processes rather than physiological load in closed-skill sports such as archery.
- Monitoring and managing mental fatigue should be prioritized in precision sports, as performance decrements may occur independently of traditional physiological markers.

**Abstract:**

**Background/Objective:** Mental fatigue (MF) significantly impairs psychomotor performance in dynamic sports; however, its specific impact on closed-skill precision-demanding tasks remains underexplored. This study investigated the acute effects of experimentally induced MF exposure on psychophysiological responses, mood states, and archery shooting performance. **Methods:** Fifteen well-trained male compound-bow archers participated in a randomized crossover study. Participants completed an MF condition (30 min modified Stroop task) and a control condition (CON; passive viewing of a neutral documentary), separated by a 72 h washout period. Continuous heart rate (HR), archery shooting accuracy, ratings of perceived exertion (RPE), rating scale of mental effort (RSME), state anxiety (VAS-A), mood states, and exercise enjoyment scale (EES) were assessed. **Results:** The Stroop task successfully induced subjective MF. Consequently, shooting accuracy significantly deteriorated in the MF condition compared to that in the CON condition (*p* < 0.001; g = 0.731). While HR and VAS-A remained consistent across conditions, the MF condition elicited a significant increase in RPE (*p* = 0.007; g = 0.836) and RSME (*p* = 0.010; g = 0.794). Furthermore, MF significantly increased feelings of anger and fatigue while drastically reducing PACES (*p* < 0.001; g = 1.530). **Conclusions:** Acute MF significantly degrades fine motor accuracy in precision sports. The pronounced dissociation between elevated RPE and stable peripheral physiological strain suggests that performance decline is driven by top-down cognitive burden rather than physiological limitations. Therefore, systematic monitoring of cognitive load is crucial for optimizing performance in precision sports.

## 1. Introduction

Prolonged cognitive activity induces mental fatigue (MF), a psychobiological state distinct from the neuromuscular limitations of physical fatigue [1,2]. In contrast to physical fatigue, which stems from neuromuscular or metabolic limitations, MF has a distinct etiology and significantly impairs physical and cognitive performance across various domains [2,3]. Theoretically, the detrimental effects of MF have been predominantly explained via the Psychobiological Model, which posits that performance is limited by an increase in the rating of perceived exertion (RPE) rather than by physiological failure [4]. However, recent neurobiological perspectives suggest that prolonged cognitive exertion involves adenosine-related neuromodulatory changes [5]. Given the critical role of the anterior cingulate cortex (ACC) in executive functions, particularly in response inhibition, sustained attention, and error monitoring, its dysfunction may adversely affect tasks that demand high levels of cognitive regulation [6]. Collectively, these mechanisms provide a plausible framework for understanding why MF may be especially detrimental in sports that depend on stable perceptual-cognitive regulation.

Most empirical evidence from ball sports indicates that MF negatively affects technical performance and tactical decision-making in open-skill sports, such as soccer [3,7]. For instance, mentally fatigued athletes demonstrate reduced accuracy in passing and shooting, slower reaction times, and impaired decision-making [3]. While the impact of MF on dynamic sports is well documented [1], the results regarding self-paced closed-skill precision tasks remain inconclusive [8]. For example, research on marksmanship has shown that while the MF impairs decision-making accuracy (shoot vs. no-shoot), it does not consistently degrade shot precision or motor stability [9]. Converging evidence [10,11] indicates that MF-related impairments in precision tasks arise not from metabolic limitations but from disruptions in attentional control and reduced suppression of task-irrelevant stimuli. Archery is a high-precision closed-skill sport that requires exceptional levels of inhibitory control and sustained attention [12,13]. Success is predicated on the ability to repeat the same shooting routine with minimal variations, a process that requires the seamless integration of visuospatial processing and fine motor control [14]. However, theoretical models suggest that MF induces mutual interference between cognitive functions and the central mechanisms driving motor behavior, resulting in increased force variability and a decline in fine motor dexterity [15,16]. Precision sports are characterized by impaired psychomotor stability, which manifests as cognitive exhaustion, increased postural sway, and impaired balance [17,18]. In archery, shooting in a difficult position increases the effects of fatigue on stability [19], and minimizing body sway is a primary determinant of achieving high scores [20], making this situation particularly important. Furthermore, recent evidence indicates that MF disrupts visual search patterns, specifically reducing quiet eye duration, which disrupts the coupling of visual attention and motor execution [21]. Consequently, the inability to maintain psychophysiological stability may lead to inconsistent clicker reaction times and reduced shooting accuracy [8,20].

Theoretically, the detrimental effects of MF on performance have been predominantly explained via the Psychobiological Model, which posits that task failure or performance degradation is driven by an accumulation of RPE rather than by direct physiological or metabolic limitations [22]. While functional neuroimaging and eye-tracking studies suggest that prolonged cognitive exertion may disrupt optimal gaze behaviors and alter executive control networks (such as the ACC), measuring these directly requires highly specialized equipment that often lacks ecological validity in field settings [23,24]. Therefore, this study examined the subjective and psychophysiological aspects of these mechanisms. Using validated perceptual scales such as the rating scale of mental effort (RSME) and RPE, this study aimed to capture the cognitive burden of archers, linking neurocognitive depletion with psychomotor performance in a simulated precision sports setting.

Experimental data on archery performance under MF conditions remain limited, despite the sport’s heavy reliance on fine motor function [8]. Furthermore, the broader literature on precision and open-skill sports yields conflicting evidence regarding athletes’ susceptibility to cognitive fatigue [25,26]. While some studies have demonstrated that elite athletes possess superior inhibitory control that buffers against fatigue [27,28], recent systematic reviews have indicated that significant impairments in psychomotor performance occur regardless of the skill level [8,29]. Therefore, the present study aimed to investigate the effects of experimentally induced MF on psychophysiological responses, mood states, and shooting performance in a simulated archery competition. Based on the psychobiological model, we hypothesized that (i) prior engagement in a cognitively demanding task would impair archery shooting performance and (ii) these performance decrements would be accompanied by elevated RPE and RSME, alongside negative shifts in affective states such as reduced enjoyment and altered mood states; (iii) reflecting altered perceptual–cognitive regulation rather than changes in physiological load.

## 2. Materials and Methods

### 2.1. Experimental Approach

This study employed a randomized crossover experimental design with two conditions: MF and control (CON). The visual analog scale (VAS), Brunel mood scale (BRUMS), and state anxiety (VAS-A) were assessed before and after each condition. In contrast, archery shooting performance, heart rate (HR), RPE, RSME, and exercise enjoyment scores (EES) were measured after task completion to evaluate the acute effects of the intervention. The crossover structure allowed each participant to serve as their own control, thereby reducing inter-individual variability and increasing statistical sensitivity. Participants completed both conditions in a counterbalanced order determined by a block randomization procedure to minimize potential order and carry-over effects. Random allocation sequences ensured that exposure to the MF and CON conditions was systematically balanced across participants. This design enabled the examination of both within-condition changes over time and condition-specific post-task responses in a simulated archery competition.

### 2.2. Participants

An a priori power analysis was conducted using G*Power (version 3.1, University of Düsseldorf, Düsseldorf, Germany) based on repeated-measures ANOVA (within factors). Assuming a medium-to-large effect size (f = 0.32), alpha level of 0.05, statistical power of 0.80, four measurements, and a correlation among repeated measures of 0.50, the required sample size was calculated to be 15 participants. The present study included 15 participants, which is considered acceptable given the within-subject crossover design. The participants were 15 well-trained male compound-bow archers (age: 20.33 ± 0.90 years; experience: 6.33 ± 0.49 years). The inclusion criteria were as follows: (i) 18–30 years of age, (ii) regular archery practice for ≥5 years, (iii) physically and psychologically healthy, and (iv) voluntary participation with written informed consent. The exclusion criteria were as follows: (i) any orthopedic, neurological, or cardiovascular disorder within the previous three months; (ii) use of psychotropic medication; (iii) cognitive impairments (e.g., vision, hearing, or attention deficits); and (iv) inability to attend the sessions regularly or complete the protocol. The study protocol was approved by the Ethics Committee of Tokat Gaziosmanpasa University (27.08.2025-614315) and conducted in accordance with the Declaration of Helsinki.

### 2.3. Measurements

Heart Rate Monitoring: HR was recorded continuously using a Polar V800 (Polar V800 Polar OY, Kempele, Finland) watch and H10 (Polar OY, Kempele, Finland) chest strap during all shooting sessions. Following the completion of the shooting protocol, the data were transferred from the Polar Flow platform to an Excel spreadsheet. The units were synchronized according to the manufacturer’s guidelines. The Polar V800 watch was placed on the right wrist, and the H10 chest strap was positioned around the chest just below the pectoral muscles. HR was recorded at one-second intervals to ensure accurate alignment with the shooting protocol timings [30].

Perceived Exertion: Shooters’ perceived exertion was measured using the RPE [6,7,8,9,10,11,12,13,14,15,16,17,18,19,20] to assess internal training intensity during each shooting session [31]. The RPE scores ranged from 6 to 20, with higher scores indicating greater perceived exertion.

Mental Fatigue: Participants were asked to draw a vertical line on a 100 mm VAS to record their subjective ratings of MF, indicating two extreme reference points at either end of the line that best represented their current experience (0 mm for “none” or no MF; and 100 mm for “extreme” MF) on a continuum [32].

Anxiety: Anxiety levels of archers were evaluated using the ‘VAS for Anxiety’ (VAS-A). The scale consists of a horizontal line measuring 100 mm, with the left end labeled ‘not anxious at all’ and the right end labelled ‘very anxious.’ The shooters were asked to mark the point on this line that most accurately reflected their current anxiety level. The VAS-A scores were determined by measuring the marked point in millimeters from the left end of the line [33].

Mood: The 24-item BRUMS assesses psychological states such as anger, confusion, depression, fatigue, tension, and vigor [34,35]. The BRUMS, which was adapted for Turkish athletes [36], was administered before and after each session to assess mood changes. Participants responded to the question ‘How are you feeling right now?’ The scale ranged from 0 (not at all) to 4 (extremely high). Using this scale allowed for a detailed assessment of how players’ moods were affected by the presence or absence of the MF.

Enjoyment: The 8-item EES, developed from the 18-item physical activity enjoyment scale (PACES) by Raedeke [37], was used to measure players’ level of EES. For this study, we used the Turkish version adapted by Soylu et al. [38], which has strong psychometric properties for adolescent and adult athletes. Participants rated their enjoyment on a Likert scale from 1 to 7, with total scores ranging from 8 (lowest) to 56 (highest).

Mental Effort: Mental effort was assessed using the RSME assessment scale, which functions as a one-dimensional tool designed to measure subjective mental effort during task performance. The mental effort assessment scale features a vertical line ranging from 0 to 150, marked with verbal indicators such as ‘no effort’, ‘some effort’, ‘moderate effort’, ‘a lot of effort’, and ‘excessive effort.’ Participants were asked to select the point on the scale that most accurately represented the mental effort expended while completing their tasks. The score was recorded as a numerical value corresponding to the point selected on the scale [39].

### 2.4. Mental Fatigue Protocol

The participants were randomly assigned to one of two conditions (MF or CON), both of which were conducted in a quiet, dimly lit environment. The MF protocol followed the procedures established in previous sports-related studies [1]. To induce MF, participants completed a paper-based modified Stroop Color-Word task, which is cognitively demanding and requires sustained attention and response inhibition [4,40]. Following a 5 min practice trial, the participants began the 30 min main task. During the task, participants were instructed to verbally report the ink color of randomly presented words (“red,” “green,” “blue,” and “yellow”), which either matched or contrasted with the written name. Under a modified rule, if a word was printed in red ink, the participants were required to read the word itself rather than state the ink color. An experienced researcher administered the session, instructing the participants to restart the sequence following any errors while encouraging them to maximize their correct responses. In the CON condition, participants passively watched an emotionally neutral nature documentary for 30 min under similar environmental conditions. They were instructed to pay attention to the screen only; no cognitive tasks or mental stimulation were provided. As demonstrated in previous studies, this passive viewing protocol does not induce MF and serves as a reliable control [1].

### 2.5. Study Design

**Figure 1:**
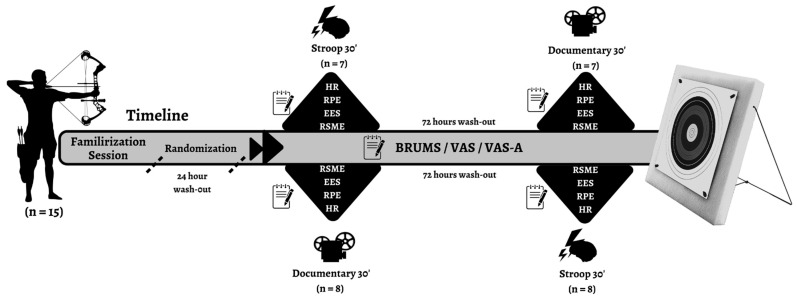
Study design.

This study employed a randomized counterbalanced crossover design to investigate the impact of MF on psychophysiological responses, mood states, and archery shooting performance. Each participant completed two experimental conditions (MF and CON) in random order. To strictly control and completely eliminate potential physiological or cognitive carry-over effects between the experimental sessions, a standardized 72 h washout period was implemented for all the participants. The temporal sequence of the assessments was rigorously structured to ensure reproducibility. Pre-intervention baseline measures of subjective VAS for MF, VAS-A, and BRUMS were administered immediately prior to the 30 min MF or CON task. Following the completion of the respective 30 min tasks, post-intervention evaluations of these psychological states were gathered instantaneously (<60 s). Subsequently, the athletes proceeded directly to the archery shooting simulation. To accurately capture the acute psychophysiological perception of the physical task without the confounding influence of rapid recovery, post-shooting parameters (RPE, RSME, and EES) were administered immediately (<120 s) following the release of the final arrow. HR was recorded continuously throughout the entire shooting phase in 1 s epochs to capture uninterrupted cardiovascular dynamics. Furthermore, all experimental sessions were conducted at the same time of day for each participant to minimize the effects of circadian rhythm. Condition sequences were randomly assigned to the participants in a balanced manner to control for any sequencing bias. All experimental sessions were conducted at the same time of day for each participant to minimize circadian rhythm effects on the results. [41]. Participants were instructed to sleep for at least 7 h before each session, avoid caffeine or stimulants in the last 6 h, and avoid strenuous physical activity in the last 24 h. Verbal encouragement was standardized throughout the testing process, and no external feedback was given. An individual archery competition environment was simulated to evaluate the participants’ shooting performance. Following calibration, the archers shot two rounds at 50 m under both conditions (MF and CON). Each round consisted of six series, and each series consisted of six arrow shots in an outdoor standard archery range (50 m). The score for each shot was determined based on the proximity of the arrow to the center of the target (x = 10 points, 9, 8, 7, 6, and 5 points). The final results of each shooting test were recorded after the simulated competition (Figure 1). Outdoor archery ballistics are highly sensitive to meteorological variables; therefore, environmental conditions were closely monitored and standardized to eliminate random variations. All experimental shooting sessions were conducted under stable weather conditions, with ambient temperatures maintained between [20 °C and 24 °C] and consistent natural daylight visibility. Crucially, wind speed was continuously monitored using a digital anemometer, and sessions were only permitted to proceed when wind speeds were registered below [2.0 m/s] to prevent non-linear lateral drift of the arrows and to minimize wind-induced postural sway of the archers [42]. The simulated competition consisted of two rounds (six series of six arrows per round) shot at a 50 m target. The total duration of the active shooting protocol was approximately (45 min) per participant. The standardized time interval ensured that the physical load remained consistent across all conditions while confirming that physiological recovery did not mask the acute effects of MF manipulation prematurely.

### 2.6. Statistically Analysis

Descriptive statistics were presented as estimated marginal means (EMMs) and standard errors (SE) derived from the models, along with arithmetic means and standard deviations (SD). Prior to the main analyses, the distribution of the data was examined using skewness and kurtosis values, and the model assumptions were evaluated based on the normality of the residuals. To appropriately account for the randomized crossover design and control for potential carry-over effects, all variables were analyzed using linear mixed-effects models (LMM). For variables measured at both pre- and post-test (BRUMS subscales, VAS, and VAS-A), the models included condition (MF and CON), time (pre-test, post-test), period, sequence, and condition × time interaction as fixed effects. For variables measured once per condition (shooting performance, HR, RPE, EES, and RSME), the models included the condition, period, and sequence as fixed effects. When significant main effects or interactions were detected, Bonferroni-adjusted pairwise comparisons of the estimated marginal means were performed to identify differences between the condition and time levels. Effect sizes for pairwise comparisons were calculated using Hedges’ g and interpreted as small (0.2 ≤ g < 0.5), medium (0.5 ≤ g < 0.8), and large (g ≥ 0.8), according to established guidelines [43,44]. For model-based analyses, partial eta-squared ($\eta_{p}^{2}$) values were reported and interpreted as small (≤0.01), medium (≥0.06), and large (≥0.14) [44,45]. Statistical analyses were conducted using SPSS (Version 27.0, IBM Corp., Armonk, NY, USA), and JASP (Version 0.19.3) was used for effect size estimation and graphical evaluation of model assumptions. Statistical significance was set at *p* ≤ 0.05.

## 3. Results

Prior to evaluating the main effects of the interventions, preliminary LMM analyses were conducted to verify the validity of the randomized crossover design. Importantly, no significant main effects of sequence or period, or any higher-order interactions involving these variables, were detected across any of the dependent psychophysiological or performance variables (all *p* > 0.05). The statistical absence of order, learning, or carry-over effects confirmed that the block randomization procedure was effective and that the strictly controlled 72 h washout period was entirely sufficient to return all parameters to baseline levels. Consequently, these factors were controlled for in the final models but were not discussed further.

**Figure 2:**
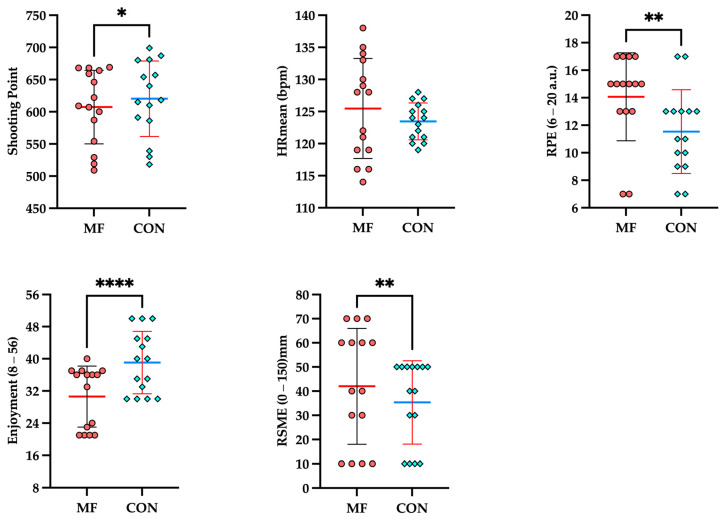
Shooting performance, RPE, EES and RSME responses under MF and CON conditions. * *p* < 0.05, ** *p* < 0.01, **** *p* < 0.0001.

Table 1 presents the EMM ± SE for psychophysiological responses and archery performance. LMM analyses revealed that the MF condition induced a significant deterioration in archery shooting accuracy compared to the CON condition (*p* < 0.05). Concurrently, the MF protocol elicited a significant amplification in RPE and RSME (*p* < 0.05), as well as a drastic reduction in EES (*p* < 0.001). Notably, despite these profound perceptual and performance decrements, HR remained completely stable across both conditions, exhibiting no statistically significant difference (*p* = 0.381) (Figure 2).

**Figure 3:**
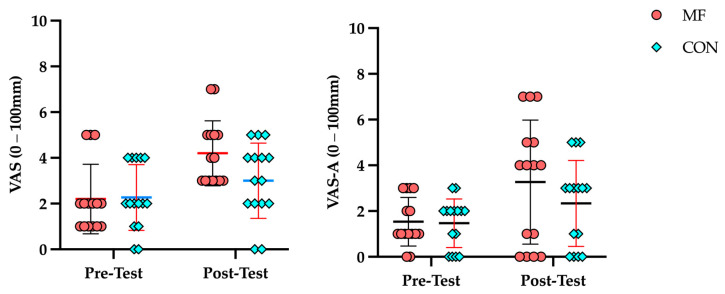
Results of VAS and VAS–A under MF and CON conditions.

The VAS and VAS-A data across conditions and time points are presented in Table 2. The LMM analysis for VAS yielded a significant condition × time interaction (*p* = 0.032), confirming that the 30 min modified Stroop task successfully induced a significantly greater magnitude in the MF condition from pre-test to post-test compared to passive documentary viewing in the CON condition. For VAS-A, a significant main effect of time was observed (*p* < 0.001), reflecting an overall increase in anxiety leading up to the shooting task; however, the condition × time interaction remained non-significant (*p* = 0.235). This indicates that the cognitive exertion protocol did not elevate VAS-A levels beyond the baseline competitive anticipation observed in the CON condition (Figure 3).

**Figure 4:**
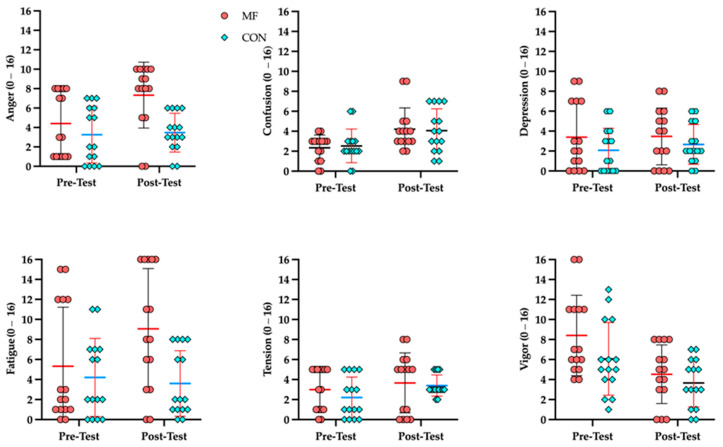
Results of BRUMS mood scale under MF and CON conditions.

The mood state responses assessed using the BRUMS are presented in Table 3. While significant main effects of time were identified for specific subscales such as Confusion (general increase, *p* = 0.001) and Vigor (general decrease, *p* = 0.001) across both sessions, the crucial Condition × Time interaction was not statistically significant for any of the six BRUMS subscales (all *p* > 0.05). These findings indicate that the magnitude of change in affective states, including anger, depression, tension, and fatigue, from pre-intervention to post-intervention did not systematically differ between the two conditions (Figure 4).

## 4. Discussion

The primary aim of this study was to investigate the impact of induced MF on archery shooting accuracy and the psychophysiological responses of archers. The data confirmed that the 30 min Stroop task successfully increased the MF perception among archers. Consequently, we observed a significant impairment in shooting accuracy under this fatigued state. In addition, a higher subjective fatigue score significantly increased the RPE and RSME scores and negatively altered mood and EES. Notably, these perceptual changes occurred without any significant influence on the HR compared with the CON condition. In summary, our results support the idea that performing an effortful cognitive task negatively influences precision performance and perception of effort, independent of physiological demand.

The current study demonstrated that MF decreases archery shooting accuracy. Because the present study relied exclusively on behavioral and psychophysiological proxies, namely RPE and RSME, rather than direct neuroimaging techniques (such as EEG, fMRI, or fNIRS), any discussion regarding the underlying cortical mechanisms remains inherently theoretical. Within an integrated neurocognitive–psychobiological framework, aiming processes rely heavily on executive functions supported by the prefrontal and cingulate cortical networks [13,41]. Prolonged engagement in cognitively demanding tasks, such as the Stroop paradigm, is thought to impair executive control efficiency [46]. Rather than drawing definitive causal conclusions regarding cortical disruption, we propose that our findings are consistent with prevailing neurocognitive models. Specifically, the observed behavioral decrements and increased RSME may reflect altered neuromodulatory processes within the ACC and frontoparietal networks, which are critical for error monitoring and response inhibition [6,12,47,48]. Furthermore, the pronounced dissociation between elevated RPE and unchanged peripheral cardiovascular strain is highly consistent with the neural efficiency hypothesis [49]. Within this theoretical framework, cognitive fatigue is hypothesized to compromise attentional stability and optimal premotor programming necessary for fine motor execution [21,50]. Consequently, reduced neural efficiency compels athletes to consciously invest greater cognitive resources to sustain motor output, which may ultimately decrease the accuracy of fine motor coordination [13,49,51]. Thus, consistent with the psychobiological model, the observed performance impairments likely emerge not from diminished peripheral motor capacity but from the increased cognitive cost of maintaining attentional regulation and visuomotor integration [2,4].

In the current study, no significant differences were observed in the HR under the present conditions. While previous studies have suggested that stable cardiovascular responses indicate that MF does not increase metabolic or autonomic demand [1,2], this conclusion must be approached with scientific caution. A fundamental statistical principle is that non-significant results do not imply the absolute absence of an effect. The regulation of HR by the autonomic nervous system is characterized by substantial inter-individual variability [52]. Given the modest sample size of this study, the statistical power required to detect subtle fluctuations in physiological variables is inherently limited, thereby increasing the probability of committing a Type II error [53]. Despite this methodological constraint, the RPE increased significantly, revealing a clear dissociation between objective physiological load and subjective effort perception. According to the psychobiological model, exercise tolerance and performance regulation are primarily governed by perceived effort rather than peripheral physiological limitations [22]. Thus, archers likely experienced the shooting task as more demanding, suggesting that MF modifies the subjective experience of effort rather than the task’s absolute physical requirements. The perceptual amplification of effort supports the notion that performance decrements originate from central nervous system mechanisms rather than from peripheral fatigue [54]. Future investigations should employ larger cohorts and integrate continuous autonomic markers, such as HR variability, to provide a more definitive physiological assessment.

The elevated RSME scores observed under MF further indicate an increase in the cognitive workload required to sustain the task performance. Precision shooting requires continuous allocation of attentional resources and fine motor monitoring [55]. Therefore, increased RSME likely reflects the compensatory recruitment of cognitive resources following executive depletion induced by prolonged cognitive activity [6,56]. Niu et al. [50] noted that this response is consistent with altered neural processing efficiency, whereby more neural activation is required to achieve similar or lower performance outcomes. MF particularly affects ACC-related performance monitoring and inhibitory processes, forcing athletes to consciously invest greater effort to maintain attentional stability and motor control [6]. Although such compensatory mechanisms may temporarily sustain performance, the increased cognitive burden ultimately compromises long-term attentional stability, thereby reducing the shooting accuracy. Therefore, heightened RSME values provide converging evidence that performance impairment is driven by cognitive resource depletion rather than by physical fatigue.

MF also influences motivational processes, as reflected by reduced enjoyment of performance. Psychobiological and motivational control models propose that sustained cognitive exertion increases the perceived cost–benefit ratio of task engagement, thereby reducing intrinsic motivation to maintain effortful performance [57]. As executive control becomes increasingly effortful, athletes may perceive a diminished reward value associated with successful task execution, leading to decrease their engagement. In precision sports such as archery, where performance relies heavily on sustained attentional investment rather than physical intensity, even subtle reductions in intrinsic motivation may negatively influence concentration and motor consistency. Consequently, decreased enjoyment likely represents a motivational consequence of increased cognitive effort under the MF condition, further contributing to performance instability.

Changes in mood responses further highlight the multidimensional impact of MF on performance. Increased feelings of fatigue and anger, along with reductions in positive affect, suggest broader psychological destabilization following cognitive exertion. Cognitive neuroscience perspectives indicate that prolonged executive engagement influences affective regulation through the shared prefrontal–cingulate circuitry involved in both cognitive control and emotional processing [51]. Such emotional alterations may disrupt the calm attentional state required for optimal performance in precision sports. Negative affective states are associated with reductions in quiet-eye duration and attentional consistency, which are key determinants of successful shooting performance [21]. Therefore, mood disturbances likely represent an additional pathway through which MF impairs performance, reinforcing the interaction among the cognitive, motivational, and emotional regulation systems in fine motor precision tasks.

The study showed that modified Stroop tests induce subjective MF, while self-reports present complications with confounding factors. The modified Stroop task elevated subjective MF, but reliance on self-reported measures requires a critical evaluation of confounding factors. The nonsignificant increase in subjective fatigue during the CON condition highlights a methodological challenge. Prolonged low-demand tasks, such as viewing a neutral documentary, can induce passive fatigue or boredom [58,59]. Subjective VAS often lack the precision to distinguish between cognitive depletion and boredom, as both manifest with similar symptoms [60,61]. Furthermore, the uniform elevation in VAS-A across both conditions suggests general arousal driven by anticipation of the archery task rather than a stress response specific to the cognitive intervention. As subjective assessments alone cannot reliably isolate MF from task-induced stress or boredom, future investigations must incorporate objective cognitive and neurophysiological indices. Measures such as electroencephalography (EEG) or pupillometry will provide more definitive validation of cognitive load and differentiate the mechanisms of mental exertion from under-arousal and anticipatory anxiety [59].

To contextualize these findings, the recent literature suggests a distinction between preserved gross motor performance and impaired fine motor control under MF. While cognitive fatigue can reduce sport-specific accuracy, such as precision tasks, it often does not affect outputs such as maximal strength or jump performance [62,63]. It has also been associated with fluctuations in sustained attention and performance, depending on the competitive context [64]. Notably, these impairments typically occur without changes in physiological markers, such as HR or lactate level [65]. The observed decrease in archery accuracy, alongside stable cardiovascular responses, is consistent with this pattern, indicating that precision-based tasks may be more sensitive to cognitive load. However, given the controlled setting and limited sample size, the findings should be interpreted cautiously, and further field-based studies are required.

### 4.1. Practical Applications

The findings of the current study have practical implications for archery training. Coaches and sports scientists should use cognitive load monitoring tools for traditional physical tracking. Daily wellness questionnaires and digital tools, such as the NASA-TLX app, combined with autonomic indicators, such as HR variability, offer a comprehensive profile of an archer’s cognitive readiness and MF vulnerability. Cognitive conditioning tools, such as 3D multiple object tracking, Neurotracker or neurofeedback training; can enhance cognitive flexibility and attentional capacity during the off-season. To counter the effects of MF during competitions, athletes should adopt pre-competition cognitive routines. Mindfulness meditation before shooting modulates cortical activity, enhancing cognitive engagement and motor precision. Rigorous pre-performance routines, such as breathing protocols, such as 6 s inhalation, 2 s hold, 7 s exhalation, and self-regulatory cueing, stimulate parasympathetic dominance. Autonomic regulation prevents the depletion of executive resources and, by suppressing anxiety, enables archers to maintain their focus and balance despite the cognitive effort required to do so.

### 4.2. Limitations

Several limitations should be acknowledged when interpreting the findings of this study. First, although the experimental protocol controlled cognitive and physiological variables, the laboratory environment may not fully replicate the perceptual and emotional demands of competitive archery, limiting ecological validity. Direct neuroimaging techniques, such as EEG, fNIRS, and fMRI, although gold standard methods, are difficult to use during compound bow shooting due to movement-related artifacts and disrupted postural dynamics [53,66]. Thus, validated psychophysiological proxies, such as the RSME and RPE, offer a practical alternative, given their associations with the prefrontal and anterior insular regions [24]. Second, the sample included only male compound-bow archers, limiting the generalizability of the findings to female athletes and other archery disciplines. Third, the 30 min Stroop task, while a method for inducing MF, may not fully reflect the cognitive stressors of real competition. Our study recorded cardiovascular responses but used the mean HR to assess the physiological load. Recent sports psychophysiology evidence shows that HR Variability (HRV), especially RMSSD and HF power, is a more sensitive autonomic marker for cognitive fatigue and parasympathetic withdrawal in precision sports than mean HR. The lack of detailed HRV analysis limits mapping autonomic nervous system dynamics and sympathetic-vagal balance during the mental fatigue protocol, indicating a methodological limitation that future research should address. Although a priori power analysis supported the sample size for primary metrics, using 15 male archers limited external validity. Studies involving elite athletes often face a “small sample dilemma,” as recruiting large, homogeneous groups is challenging [53]. Although the randomized crossover design reduces inter-individual variability, small samples may produce wider confidence intervals and lower power to detect small-to-medium effects, especially for variable measures, such as HR [67]. Thus, the findings should be considered preliminary. Hedges’ g was used as a conservative effect size estimate to address potential sample size bias. Future large-scale longitudinal studies with diverse athlete populations, including female archers, are required to enhance ecological validity.

## 5. Conclusions

In conclusion, this study demonstrated that 30 min of intensive cognitive exertion induces a state of MF that significantly impairs archery accuracy. This impairment is characterized by an increased perception of effort and disruption of the psychophysiological readiness required for high precision motor tasks. These findings suggest that the decline in performance is driven by central mechanisms, specifically a rise in the perceived cost of focus and a reduction in neural efficiency, rather than peripheral physiological limitations. For practitioners and coaches, these results emphasize the importance of monitoring athletes’ daily cognitive loads. Furthermore, as discussed, future research should explore the efficacy of cognitive conditioning interventions, such as brain endurance training, in mitigating these detrimental effects in competitive environments.

## Figures and Tables

**Table 1:** EMM for shooting performance and psychophysiological responses under mental fatigue and control conditions.

	MF EMM ± SE	CON EMM ± SE	Mean Difference	%95 CI		ES (g)	F	*p*
				Lower	Upper			
Shooting (point)	606.71 ± 41.10	619.62 ± 41.10	−12.91	−22.91	−2.91	−0.73	7.78	**0.015**
HR (beat·min^−1^)	125.52 ± 2.13	123.53 ± 2.13	1.99	−2.76	6.74	0.21	0.82	0.381
RPE (6–20 a.u.)	14.07 ± 1.02	11.53 ± 1.02	2.54	0.84	4.25	0.84	10.36	**0.007**
EES (8–56)	30.69 ± 2.01	39.20 ± 2.01	−8.51	−11.61	−5.41	−1.53	35.16	**<0.001**
RSME (0–150 mm)	41.88 ± 5.59	35.27 ± 5.59	6.61	1.90	11.31	0.79	9.20	**0.010**

Note. MF = Mental fatigue; CON = Control; EMM = Estimated Marginal Mean; SE = Standard Error; CI = Confidence Interval for the mean difference (MF − CON); ES = Effect Size (Hedges’ g); HR = Heart rate; RPE = Rating of perceived exertion; EES = Exercise enjoyment scores; RSME = Rating Scale of mental effort. The F statistics reflect the main effect of the condition from the Linear Mixed Models. Bold *p*-values indicate statistical significance (*p* ≤ 0.05).

**Table 2:** EMM for VAS and VAS-A across time and conditions.

	MF EMM ± SE [95% CI]		CON EMM ± SE [95% CI]		F (Interaction)	*p*
	Pre	Post	Pre	Post		
VAS (mm)	2.18 ± 0.39 [1.38, 2.98]	4.19 ± 0.42 [3.32, 5.06]	2.47 ± 0.39 [1.67, 3.27]	3.21 ± 0.42 [2.34, 4.08]	4.96 (1, 39.4)	0.032
VAS–A (mm)	1.52 ± 0.33 [0.82, 2.21]	3.23 ± 0.50 [2.22, 4.25]	1.54 ± 0.33 [0.85, 2.23]	2.48 ± 0.50 [1.47, 3.49]	1.47 (1, 28.9)	0.235

Note. MF = Mental fatigue; CON = Control; EMM = Estimated Marginal Mean; SE = Standard Error; VAS = Visual Analog Scale; VAS–A = Visual Analog Scale–Anxiety. F and *p*-values represent the Condition × Time interaction from the Linear Mixed Models. *p*-values indicate statistical significance (*p* ≤ 0.05).

**Table 3:** Estimated marginal means for the BRUMS mood scale under mental fatigue and control conditions.

	MF EMM ± SE [95% CI]		CON EMM ± SE [95% CI]		F	*p*
	Pre-Test	Post-Test	Pre-Test	Post-Test		
Anger (0–16)	4.40 ± 3.29 [2.21, 5.66]	7.33 ± 3.39 [3.91, 7.42]	3.27 ± 2.91 [2.10, 5.55]	3.47 ± 2.00 [3.45, 6.97]	0.05	0.827
Confusion (0–16)	2.33 ± 1.29 [−0.20, 5.14]	4.20 ± 2.14 [0.28, 8.00]	2.53 ± 1.68 [−0.21, 4.90]	4.07 ± 2.19 [0.31, 7.85]	0.01	0.937
Depression (0–16)	3.40 ± 3.42 [1.24, 4.43]	3.47 ± 2.85 [1.85, 4.37]	2.07 ± 2.22 [1.03, 4.23]	2.67 ± 1.99 [1.65, 4.15]	0.00	0.995
Fatigue (0–16)	5.33 ± 5.89 [2.27, 7.64]	9.07 ± 6.02 [3.87, 9.73]	4.20 ± 3.90 [1.97, 7.34]	3.60 ± 3.27 [2.81, 8.67]	0.08	0.779
Tension (0–16)	3.00 ± 2.10 [1.53, 3.83]	3.67 ± 2.99 [2.39, 4.70]	2.20 ± 2.04 [1.35, 3.66]	3.40 ± 1.06 [2.25, 4.56]	0.00	0.975
Vigor (0–16)	8.40 ± 4.01 [5.34, 9.30]	4.53 ± 2.92 [2.44, 5.32]	6.07 ± 3.63 [4.81, 8.81]	3.67 ± 2.41 [2.75, 5.64]	0.23	0.634

Note. MF = Mental fatigue; CON = Control; EMM = Estimated Marginal Mean; SE = Standard Error. F and *p*-values represent the Condition × Time interaction effect from the Linear Mixed Models. The effect sizes for non-significant interactions were omitted as they were negligible.

## Data Availability

The data supporting the findings of this study are available from the corresponding author upon reasonable request. Owing to ethical restrictions and participant confidentiality, the data are not publicly available.

## Abbreviations

The following abbreviations are used in this manuscript:

MF	Mental fatigue
CON	Control
RPE	Rating of perceived exertion
VAS	Visual analog scale
VAS-A	Visual analog scale-Anxiety
RSME	Rating scale of mental effort
EES	Exercise enjoyment scores
BRUMS	Brunel mood scale
HR	Heart rate
ACC	Anterior cingulate cortex
ES	Effect size
SD	Standard deviation
QE	Quiet-eye
